# Micheliolide prevents estrogen deficiency-induced bone loss via inhibiting osteoclast bone resorption

**DOI:** 10.18632/aging.205111

**Published:** 2023-10-11

**Authors:** Ziyang Gan, Junming Huang, Mingyou Xu, Xingshi Yuan, Xifu Shang, Xi Chen, Kun Chen

**Affiliations:** 1 Department of Orthopedics, The First Affiliated Hospital of USTC, Division of Life Sciences and Medicine, University of Science and Technology of China, Hefei 230001, Anhui, China; 2 Department of Orthopedics, The First Affiliated Hospital of Nanchang University, Nanchang 330000, Jiangxi, China

**Keywords:** osteoporosis, osteoclast, osteoblast, MAPK, Micheliolide

## Abstract

Osteoporosis is one of the major health problems characterized by decreased bone density and increased risk of fractures. Nowadays, the treating strategies against osteoporosis are efficient, but still have some drawbacks. Micheliolide, a guaianolide sesquiterpene lactone isolated from Michelia compressa and Michelia champac, has been reported to have anti-inflammatory effects. Here, our data suggest that Micheliolide could protect mice from ovariectomy induced bone loss. According to the Micro-CT scan and histomorphometry quantification data, Micheliolide treatment inhibits excessive osteoclast bone resorption without affecting bone formation in estrogen deficiency mice. Consistently, our data suggest that Micheliolide could inhibit osteoclastogenesis *in vitro.* Additionally, we confirmed that Micheliolide inhibits osteoclasts formation via inhibiting P38 MAPK signaling pathway, and P79350 (a P38 agonist) could rescue this effect. In summary, our data suggest that Micheliolide could ameliorate estrogen deficiency-induced bone loss via attenuating osteoclastogenesis. Hence, Micheliolide could be used as a novel anti-resorptive agent against osteoporosis.

## INTRODUCTION

Osteoporosis is a metabolic bone disease caused by the dysregulation of osteoclast and osteoblast [1, 2]. It has been recognized as one of the major health problems affecting more than 200 million people worldwide, leading to heavy clinical and economic burden [3]. Statistically, females are more likely to develop osteoporosis than males as a result of estrogen depletion during menopause. Presumably, 20.6% women aged 40 years or older suffer from osteoporosis in China [4]. Thus, the prevention and treatment of osteoporosis, especially postmenopausal osteoporosis, will undoubtedly become more urgent due to due to the aging population.

In recent years, a number of drugs have been discovered for treating osteoporosis. These drugs could be divided into two types: one is anti-resorptive drugs which function via inhibiting osteoclast bone resorption, including bisphosphonates, selective estrogen receptor modulators (SERMs), RANKL antibodies and so on; another one is anabolic drugs which enhance osteoblast bone formation, including Teriparatide and abaloparatide [5]. These agents are efficient, but far from ideal [6]. As is reported, teriparatide might cause nausea, headache and is associated with the increasing risk of osteosarcoma [7–9]. Bisphosphonates, one of the most widely used anti-resorptive agents, has been reported to have side-effects such as osteonecrosis of the jaw and atypical fractures. Recently, osteocytes were demonstrated as a key factor in bone metabolism through regulating both osteoblast and osteoclast. In 2019, romosozumab, which targets osteoclastic sclerostin, was approved for treating osteoporosis in postmenopausal women, but associated cardiovascular risks limit its use (29931216). So, it is still imperative to find new drugs with little side effects treating osteoporosis [10–12].

Micheliolide (MCL) is a guaianolide sesquiterpene lactone isolated from Michelia compressa and Michelia champaca [13, 14]. In recent years, more and more studies suggest that MCL has therapeutic effects on inflammatory diseases and tumors, including rheumatoid arthritis, acute myelogenous leukemia, inflammatory bowel diseases and so on [14–16]. In a previous study, MCL inhibits doxorubicin-induced cardiotoxicity by inhibiting PI3K/Akt/NF-kB signaling, alleviating inflammation and necrosis (30835049). Furthermore, MCL has been shown to induce an increase in peroxisome proliferator-activated receptor-1 expression, thus alleviating NF-B-mediated inflammation and enhancing autophagy. However, to the best of our knowledge, it is still unclear whether Micheliolide had an effect on osteoporosis. Our current work aims to study the effects of Micheliolide on estrogen deficiency induced osteoporosis and to discover how Micheliolide exert its effects.

## RESULTS

### Micheliolide protected mice from OVX induced bone loss

**Figure 1:**
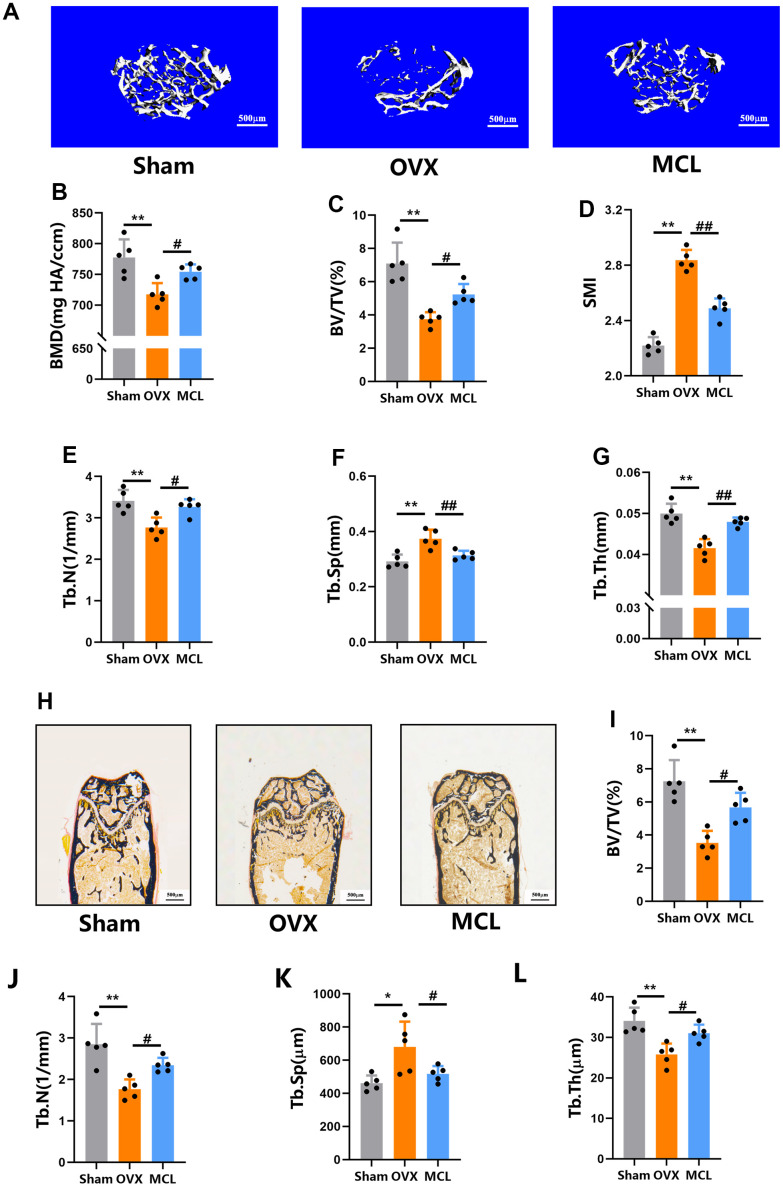
**Micheliolide protected mice from OVX induced bone loss.** Fifteen wild type female C57/BL6 mice aged 8 weeks were obtained and randomly split into three groups (n=5): Sham group (with Sham surgery and following with vehicle treatment), OVX group (with OVX surgery following with vehicle treatment), MCL group (with OVX operation following with Micheliolide treatment (30 mg/kg/day)). (**A**) Representative 3D micro-CT images of proximal femur. Scale bar = 500 μm. (**B**–**G**) Micro-CT values of BMD (mg HA/ccm), BV/TV (%), SMI, Tb.N (1/mm), Tb.Th (mm), and Tb.Sp (mm) were analyzed using the built-in software. (**H**) Representative Von Kossa staining of the histomorphological sections from each indicated group. (**I**–**L**) BV/TV, Tb.Th, Tb.N, and Tb.Sp of the Von Kossa staining sections from each group. All data: mean ± SD, n=5, *p < 0.05 comparing to Sham group, **p < 0.01 comparing to Sham group, # p < 0.05 comparing to Sham group, ## p < 0.01 comparing to Sham group.

In order to determine the effect of Micheliolide on osteoporosis, OVX mice model, one of the most sophisticated animal models for osteoporosis research, was used. In short, mice were randomly divided into 3 groups with different treatments: 1) Sham group, mice undertaken sham surgery and following with saline treatment; 2) OVX group, mice undertaken OVX surgery and following with saline treatment; 3) Micheliolide group, mice undertaken OVX surgery and following with Micheliolide (30 mg/kg) treatment according to previous study [17]. After 6 weeks of treatment, all mice were collected and the bone mass was analysis. As expected, “OVX group” showed a significant drop of bone mass compared to “Sham group”, confirming the successful construction of the OVX model (Figure 1).

Notably, micro-CT data showed that BV/TV, BMD, SMI, Tb.N and Tb.Th in “Micheliolide group” elevated significantly compared to “OVX group” (Figure 1A–1E, 1G). Meanwhile, Tb.Sp dropped in “Micheliolide group” (Figure 1F). These micro-CT data suggested that the treatment of Micheliolide ameliorated OVX induced bone loss in mice.

To further confirm that, we also conducted histomorphometry analysis. Consistently, the histomorphological quantification data also showed a dramatic rise in BV/TV, Tb.N, Tb.Th in “Micheliolide group” compared to “OVX group” (Figure 1H–1L), reconfirming that the administration of Micheliolide could protect mice from estrogen-deficiency induced osteoporosis.

### Micheliolide attenuated OVX induced excessive osteoclast bone resorption without affecting bone formation

**Figure 2:**
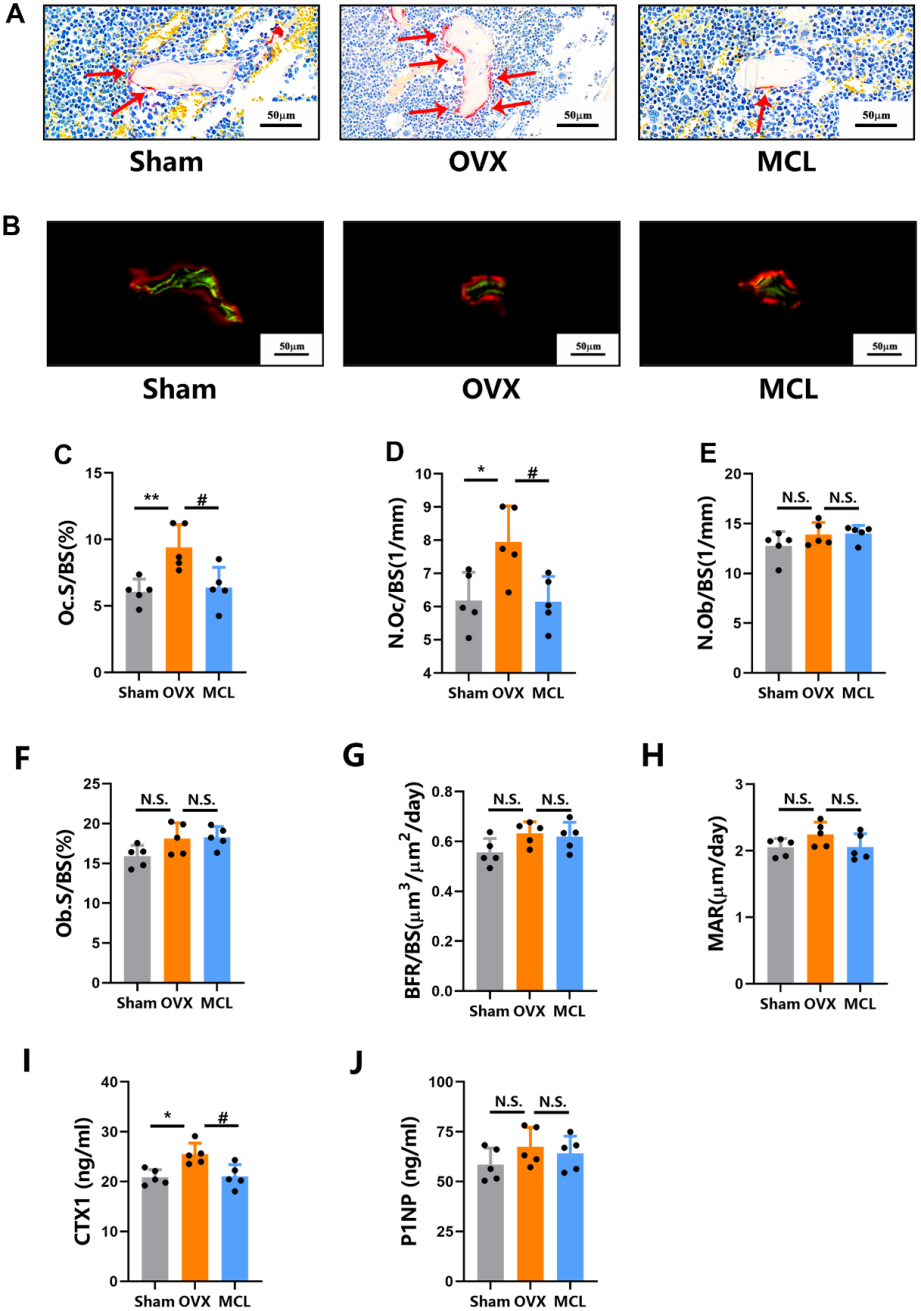
**Micheliolide attenuated OVX induced excessive osteoclast bone resorption without affecting bone formation.** Fifteen wild type female mice were randomly split into 3 groups (Sham group, OVX group and MCL group) as described in Figure 1. (**A**) Representative images of the plastic section TRAP staining pictures. Red arrows demonstrate stained TRAP positive osteoclasts. Scale bar = 50um. (**B**) Representative pictures of calcein (green) and alizarin red (red) labels were visualized. Scale bar = 50um. (**C**, **D**) The OcS/BS and N.Oc/BS were measured by TRAP staining sections. (**E**, **F**) N.Ob/SB and ObS/BS were measured by Toluidine Blue staining sections. (**G**, **H**) BFR/BS and MAR were measured by undecalcified sections. (**I**, **J**) The serum level of CTX1 and P1NP were evaluated. All data: mean ± SD, n=5, *p < 0.05 comparing to Sham group, **p < 0.01 comparing to Sham group, # p < 0.05 comparing to Sham group, ## p < 0.01 comparing to Sham group, N.S. no significant difference between each group.

To further study the mechanism by which Micheliolide protects mice from bone loss, the bone metabolism status was evaluated. As expected, ovariectomy induced excessive activation of osteoclasts without significant bone formation changes, as shown by the increased Oc.S/BS, N.Oc/BS and no significant differences of N.Ob/BS, Ob.S/BS, BFR/BS, MAR in OVX group compared to sham group (Figure 2A–2H). Importantly, we observed that Oc.S/BS and N.Oc/BS in Micheliolide group dropped significantly compared to OVX group, suggesting that the administration of Micheliolide attenuated osteoclast bone resorption (Figure 2A, 2C, 2D). On the other hand, no significant differences of bone formation parameters (N.Ob/BS, Ob.S/BS, BFR/BS and MAR) were observed between Micheliolide group and OVX group, indicating that the treatment of Micheliolide didn’t affect bone formation status (Figure 2E–2H).

Additionally, we also evaluated the mice serum bone metabolism markers P1NP and CTX-1 [18]. As is shown, no significant differences were observed for bone formation marker P1NP levels between three groups, indicating no changes in bone formation status after Micheliolide treatment. Meanwhile, OVX group exhibited much higher CTX-1 levels compared to Sham group. And the Micheliolide group showed significantly decreased CTX-1 levels comparing to OVX group. Considering that CTX-1 is an important bone resorption marker, this data suggested the inhibition effect of Micheliolide on bone resorption (Figure 2I, 2J). Taken together, these data demonstrated that the administration of Micheliolide attenuated mice bone resorption without affecting bone formation.

### Micheliolide inhibited osteoclastogenesis without any cytotoxicity

**Figure 3:**
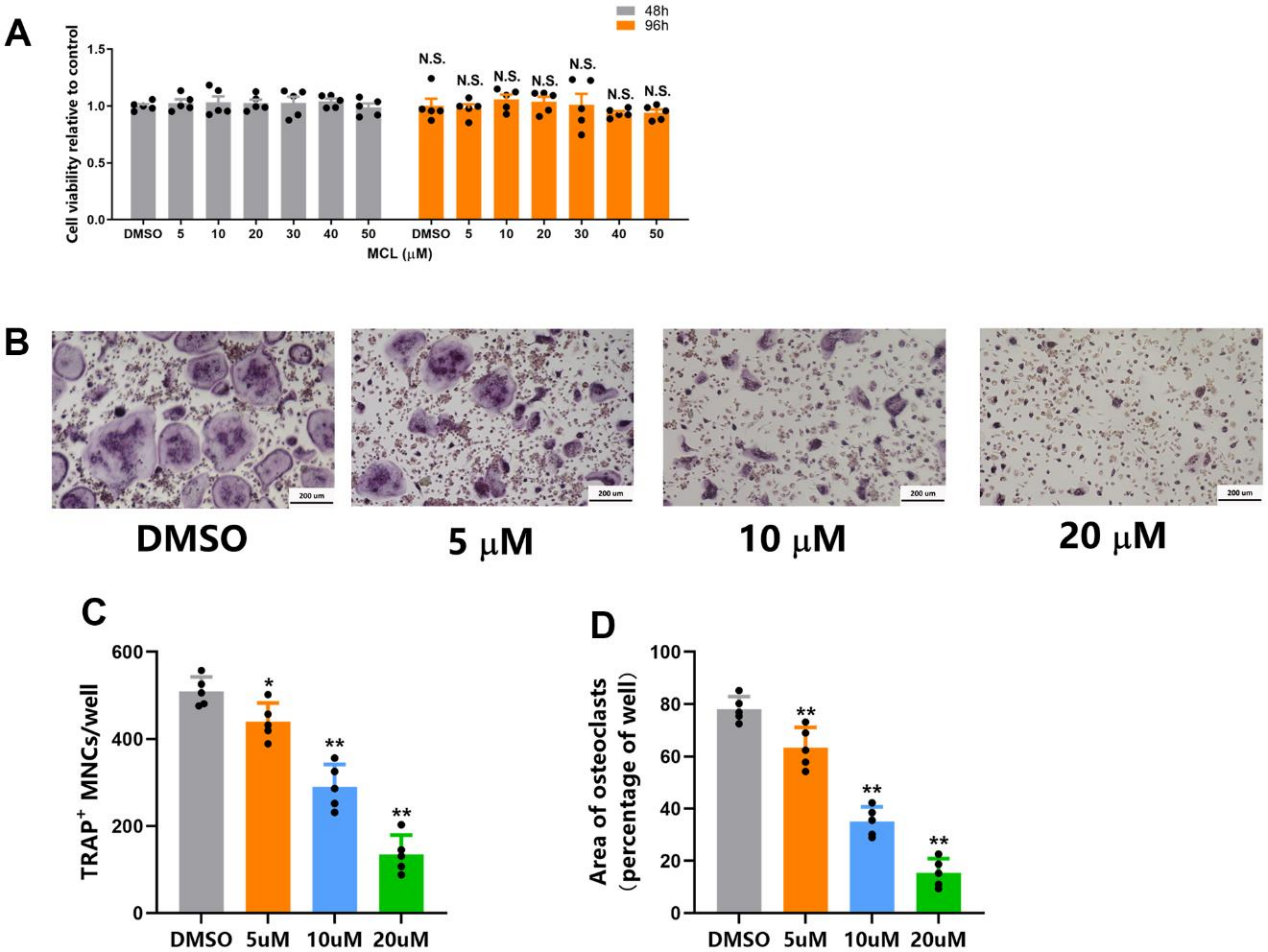
**Micheliolide inhibited osteoclastogenesis without any cytotoxicity.** (**A**) BMMs were seeded on 96-well plates at a density of 1.5*10^4^ cells/well. Then cells were treated with vehicle or different concentrations (5 ug/ml, 10 ug/ml, 20 ug/ml) of Micheliolide for 48 or 96 hours. The cell viability was measured by CCK-8 kit. (**B**) Representative TRAP staining images from BMMs treated with vehicle or different concentrations (5 ug/ml, 10 ug/ml, 20 ug/ml) of Micheliolide in osteoclastogenesis medium for 5 days. Scale bar = 200 um. (**C**) Numbers of TRAP positive multinuclear osteoclasts from different groups. (**D**) Area of TRAP positive multinuclear osteoclasts from different groups. All data: mean ± SD, n=5, N.S. no significant difference between each group.

Osteoclasts are the principal, if not exclusive, bone resorption cells [19]. Considering that Micheliolide inhibits bone resorption in OVX mice*,* we next look at the effects of Micheliolide on osteoclasts *in vitro*. Firstly, the cytotoxicity assays were performed. Bone marrow macrophages (BMMs) were treated with vehicle (DMSO) or different doses of Micheliolide (5, 10, 20, 30, 40, 50 μM) for 24 or 48 hours. The data suggested that Micheliolide didn’t have any toxicity on BMMs even at a relatively high dose (50 μM) (Figure 3A). Next, BMMs were stimulated with M-CSF and RANKL to induce osteoclast differentiation. As is shown by TRAP staining and the quantification results, Micheliolide inhibited osteoclastogenesis in a dose dependent manner (Figure 2B–2D). These results manifested that Micheliolide inhibited osteoclast differentiation without any cytotoxicity.

### Micheliolide inhibited osteoclast bone resorption and related gene expressions

**Figure 4:**
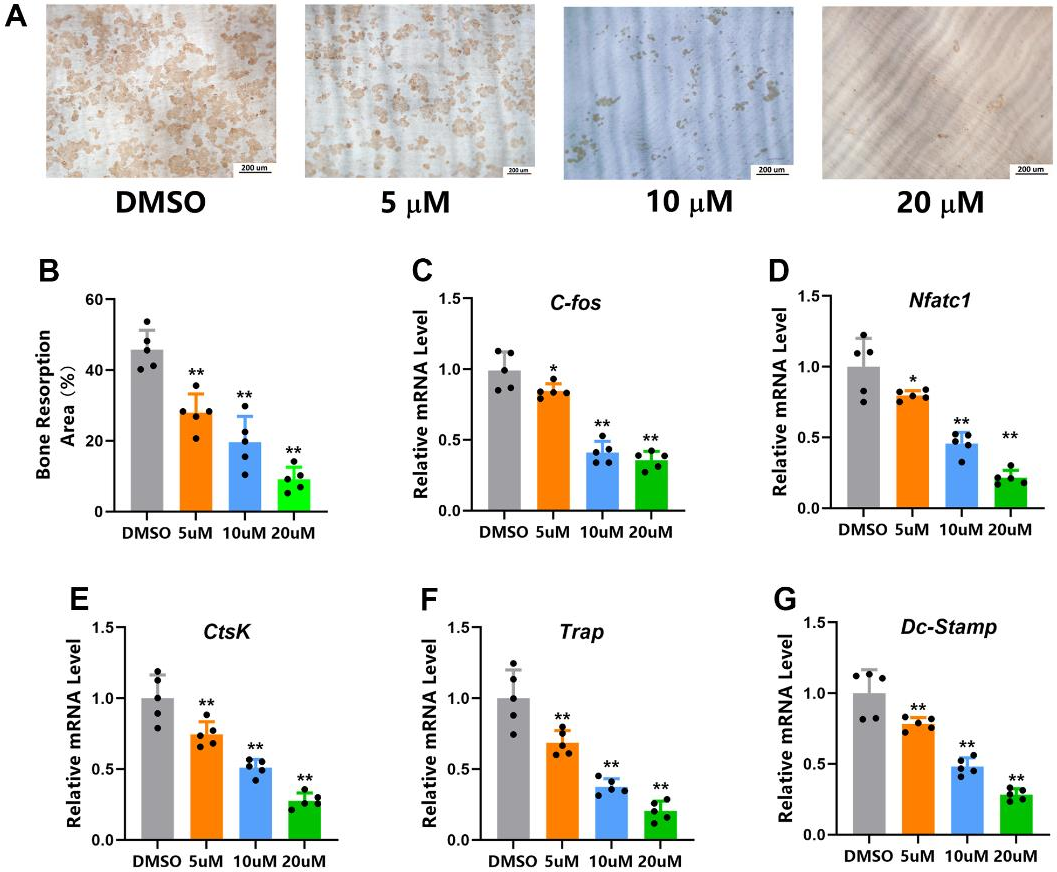
**Micheliolide inhibited osteoclast bone resorption and related gene expressions.** (**A**) Representative images of bone resorption pits. (**B**) Bone resorption area (brown area) was measured. (**C**–**G**) mRNA expression of *C-fos, Nfatc-1*, *CtsK*, *Trap* and *Dc-Stamp* of mice BMMs received treatment of indicated doses of Micheliolide (5 ug/ml, 10 ug/ml, 20 ug/ml) or vehicle in the presence of M-CSF and RANKL for 5 days. All data: mean ± SD, n=5, *p < 0.05 comparing to Sham group, **p < 0.01 comparing to DMSO group.

We next performed pit assays to evaluate the effects of Micheliolide on osteoclast function. First of all, mature osteoclasts were obtained via seeding BMMs on collagen I coated plates together with M-CSF and RANKL for 5 days. After that, mature osteoclasts were digested and reseeded on dentin slices, continued treating with M-CSF and RANKL together with Micheliolide (20 μM) or DMSO for another 2 days. Then, dentin slices were collected, and the bone resorption area was evaluated. Notably, as is shown in Figure 4, Micheliolide inhibited osteoclast bone resorption in a dose dependent manner (Figure 4A, 4B).

In order to further confirm the effects of Micheliolide on osteoclasts, RT-PCR of osteoclast related genes including *Trap*, Cathepsin K (*Ctsk*), dendritic cell-specific transmembrane protein (*DC-STAMP*), *C-fos* and nuclear factor of activated T cells c1 (*NFATc1*) were tested. As is shown in figure 4, Micheliolide treatment (5, 10, 20 μM) exhibited an inhibition effect on these genes (Figure 4C–4G). Together, these results showed that Micheliolide inhibited osteoclast bone resorption and related gene expressions.

### Micheliolide inhibited RANKL-induced activation of the p38 MAPK signaling pathway during osteoclast differentiation

**Figure 5:**
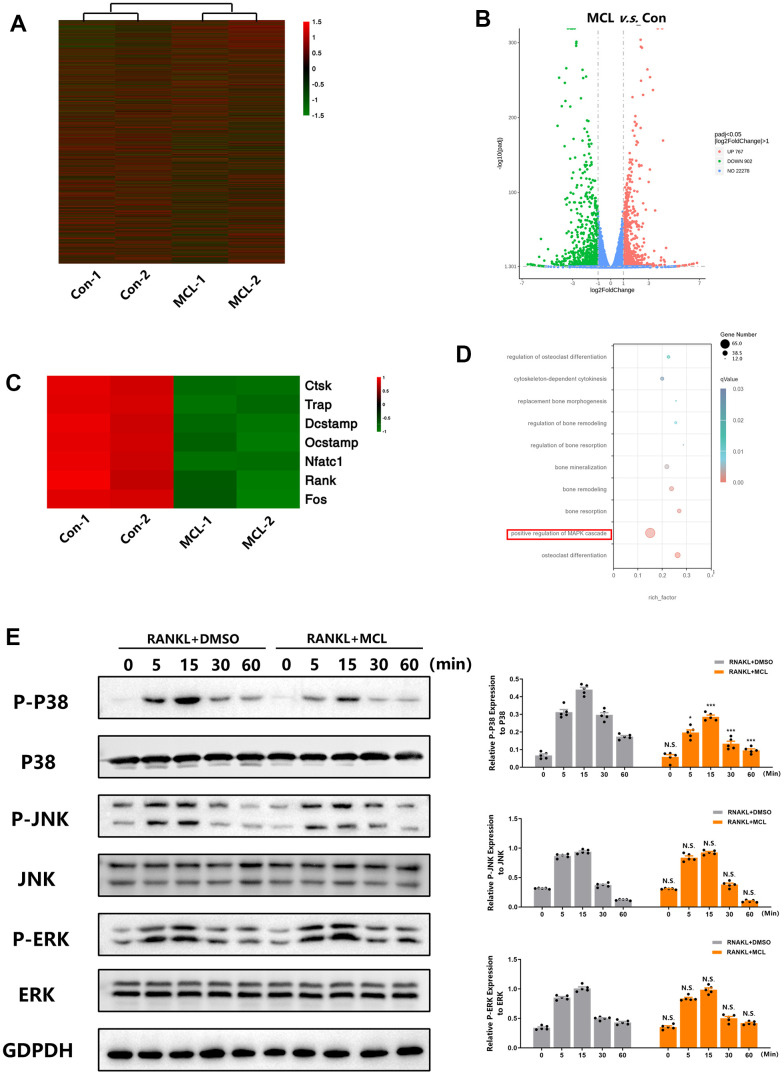
**Micheliolide inhibited RANKL-induced activation of the p38 MAPK signaling pathway during osteoclast differentiation.** BMMs were plated at the same density of 2.5*10^3^ cell/cm in 4 different wells. 2 wells received DMSO treatment (Con-1, Con-2) and another 2 wells received 20 uM Micheliolide (MCL-1, MCL-2) treatment in the presence of M-CSF and RANKL for 5 days. Then, samples were collected and sent for RNA-seq. (**A**) Heatmap of the total 23947 detected genes in control and Micheliolide treatment group. (**B**) Volcano Plot of the detected genes. (**C**) Heatmap of osteoclast related genes detected in control and Micheliolide treatment group. (**D**) GO analysis of the RNA-sequencing. (**E**) BMMs were pre-treated with DMSO or Micheliolide (20 uM) for 2 hours, then stimulated with RANKL for indicated times (0, 5, 15, 30, 60 min). Proteins were collected and WB for MAPK signaling pathway was performed. All data: mean ± SD, n=5, *p < 0.05 comparing to RANKL+DMSO group, **p < 0.01 comparing to RANKL+DMSO group, ***p < 0.001 comparing to RANKL+DMSO group, N.S. no significant difference comparing to RANKL+DMSO group.

The aforementioned results promoted us to study how Micheliolide regulates osteoclasts formation and function. RNA-seq was utilized to evaluate the gene expressions in osteoclasts with or without Micheliolide (20 μgM) treatment. In total, 23947 genes were detected in control and Micheliolide treatment group, and 1669 genes were considered as differentially expressed genes (DEGs) (Figure 5A). Among these genes, 902 genes were significantly down-regulated, while another 767 genes were dramatically elevated in Micheliolide treatment group compared to control (Figure 5B). Of note, osteoclast related genes including *Ctsk*, *Trap*, *Dcstamp*, *Ocstamp*, *Nfatc1*, *Rank*, and *C-fos* were considerably downregulated in Micheliolide treatment group (Figure 5C). This result is in accordance with the aforementioned RT-PCR data (Figure 4C–4G), reconfirming the inhibitory effect of Micheliolide on osteoclast differentiation and function. Moreover, the GO analysis indicated that many osteoclast-related biological processes such as “osteoclast differentiation”, “bone resorption”, “bone remodeling”, “bone mineralization” and “replacement bone morphogenesis” were enriched among the DEGs in Micheliolide treated cells (Figure 5D). Importantly, we noticed that MAPK signaling pathway was enriched as well, indicating the possibility that Micheliolide might affect MAPK signaling pathway in osteoclasts (Figure 5D). It has been established that MAPK signaling pathway is one of the most important signaling pathways involved during osteogenesis. Western blot (WB) was performed to further validate the effect of Micheliolide on MAPK signaling. Impressively, WB data revealed that P38 signaling was strongly suppressed while ERK and JNK signaling were not affected under Micheliolide treatment (Figure 5E).

### The p38 agonist rescued the inhibitory effect of Micheliolide on osteoclast differentiation

**Figure 6:**
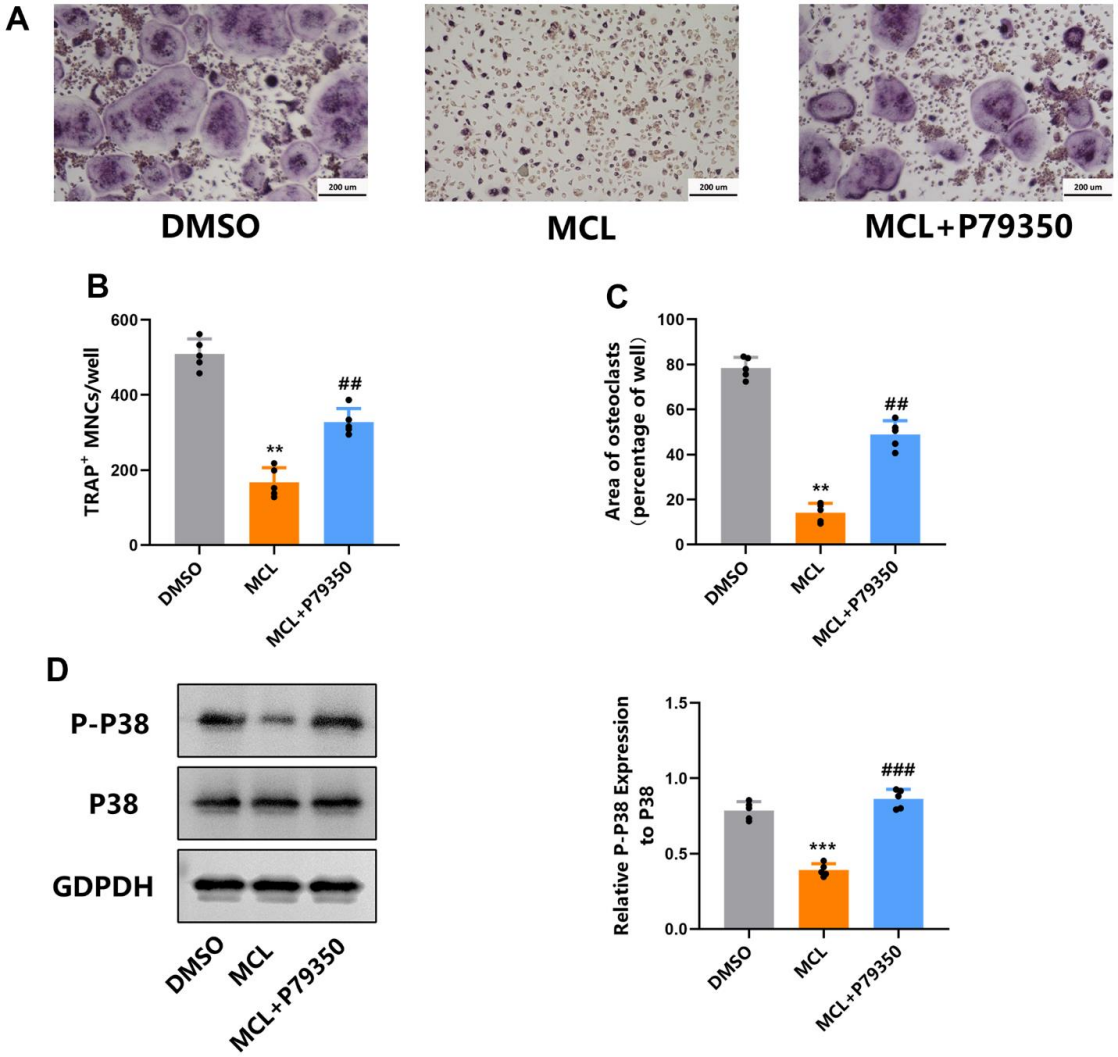
**The p38 agonist partly rescued the inhibitory effect of Micheliolide on osteoclastogenesis.** BMMs were isolated, treated with “DMSO” or “MCL” (20 uM) or “Micheliolide and P79350” in each group together with M-CSF and RNAKL for 5 days. (**A**) Representative TRAP staining pictures. (**B**, **C**) Number and area of osteoclasts from each group were measured and quantified. (**D**) WB for P-P38, P38 and GAPDH was performed. All data: mean ± SD, n=5, **p < 0.01 comparing to DMSO group, ***p < 0.001 comparing to DMSO group, ##p < 0.01 comparing to MCL group, ###p < 0.001 comparing to MCL group.

As is mentioned above, Micheliolide prohibited the activation of p38 signaling during osteoclast differentiation. This gives us a hint that Micheliolide might could exert its osteoclast inhibitory effect via suppressing p38 signaling. In order to prove this hypothesis, P79350, a kind of p38 agonist, was used to treat osteoclast together with Micheliolide during osteogenesis. WB data showed that P79350 significantly increased p-p38 levels in “MCL+P79350” group compared to “MCL” group, confirming the successful activation of p38 signaling (Figure 6D). Notably, compared to the Micheliolide treated group, the Micheliolide and P79350 co-treated group formed more osteoclasts, supported by the dramatically elevated osteoclasts numbers (Figure 6A–6C). In all, these results demonstrated that p38 agonist reversed the inhibitory effect of Micheliolide on osteoclastogenesis, proving the hypothesis that Micheliolide inhibited osteoclastogenesis via prohibiting p38 MAPK signaling.

## DISCUSSION

Osteoporosis is a bone metabolism disease caused by the imbalance between osteoclast bone resorption and osteoblast bone formation, characterized by decreased BMD and increased risk of fragility fractures [20]. Based on the development of the disease, osteoporosis is typically divided into two main types: primary osteoporosis and secondary osteoporosis. Postmenopausal osteoporosis caused by estrogen decline is the most prevalent types of osteoporosis and has been recognized as one of the major health problems which affects more than 200 million individuals, causing heavy clinical and economic burden [21]. As far back as the 1950s, estrogen was suggested as a pharmacological remedy for osteoporosis because of its ability to hinder osteoclasts. However, the utilization of estrogen is associated with the possibility of developing endometrial and breast cancer. In recent years, a number of drugs have been discovered for treating osteoporosis. These drugs could be divided into two types: one is anti-resorptive drugs which function via inhibiting osteoclast bone resorption, including bisphosphonates, selective estrogen receptor modulators (SERMs), RANKL antibody and so on. Of these drugs, bisphosphonates serve as the primary choice of medication for decreasing the loss of bone density, but prolonged use can bring about concerns about atypical femoral fractures and osteonecrosis of the jaw (31523544). Another one is anabolic drugs which enhance osteoblast bone formation, including Teriparatide and abaloparatide. The anabolic agents were only recommended to treat patients at high risk for fracture because of potential increase in the risk of osteosarcoma [22–25]. It is important to mention that romosozumab, targeting sclerostin secreted by osteocytes, belongs to a new class of medication that has a “dual effect” of increasing bone formation while decreasing bone resorption. Unfortunately, concerns regarding a potential risk of cardiovascular disease were persisted (36382760). Although these agents are effective, they are far from being ideal, promoting us to find new effective drugs with little side effects for osteoporosis.

Micheliolide is colorless crystal, stable in blood. MCL has been reported to inhibit rheumatic arthritis, intestinal inflammation and many other diseases [14, 26, 27]. In spite of the therapeutic potential shown in these studies, MCL has not been reported as a treatment for osteoporosis. For this reason, we conducted this study to evaluate the efficacy of MCL in treating osteoporosis, especially postmenopausal osteoporosis. In the present study, we aim to discover if Micheliolide could have some therapeutic effects on osteoporosis. Firstly, we constructed OVX mice model, testing the effects of Micheliolide *in vivo*. Instructively, Micheliolide ameliorates estrogen deficiency induced bone loss, supported by the dramatically increased BV/TV after Micheliolide treatment (Figure 1). Meanwhile, the histomorphometry assays demonstrated that Micheliolide attenuated OVX-induced excessive osteoclast bone resorption (Figure 2), promoting us to further study the effects of Micheliolide on osteoclasts. Impressively, Micheliolide strongly inhibited osteoclast formation and function, supported by the significantly decreased osteoclast formation number, largely reduced bone resorption pit area and dramatically down-regulated osteoclast related genes with the treatment of Micheliolide (Figures 3, 4). Hence, based on our results, Micheliolide exerted a strong inhibitory effect on osteoclast formation and function both *in vivo* and *in vitro*.

The major concern for the current anti-resorptive drugs against osteoporosis is that the bone resorption inhibitory effect is often combined with the inhibition of bone formation because of the coupling of these two processes [28]. For example, bisphosphonates, one of the most widely used anti-resorption drugs, have been reported to have side effects causing osteonecrosis of jaw and atypical fractures due to its inhibitory effect on bone formation [29–31]. So, drugs which could inhibit osteoclast function without affecting osteoblast bone formation are more desirable. Notably, Micheliolide meets this requirement. According to histomorphometry assays, no significant differences of bone formation parameters including N.Ob/BS, ObS/BS, MAR, BFR/BS and serum P1NP levels were observed with or without Micheliolide treatment, indicating that Micheliolide didn’t affect bone formation status (Figure 2). Together, our data demonstrate that Micheliolide could prohibit bone resorption and do not affect bone formation which suggests that Micheliolide could be a promising drug for osteoporosis.

We also evaluated the underlying mechanism by which Micheliolide exerted its effects on osteoclasts. A number of signaling cascades have been reported to be associated with osteoclast, including MAPK, AKT, NF-κB signaling and so on [32]. Among these various signaling cascades, p38 MAPK signaling has been considered as one of the most important for osteoclast differentiation and function [33]. In osteoclastogenesis, activated p38 directly phosphorylates and stimulates NFATc1 and MITF, transcription factors that induce osteoclastic proteins, such as TRAP, cathepsin K, and E-cadherin (11792706 15304486). In addition, RANKL-stimulated active p38 leads to the phosphorylation of the NF-κB p65 subunit at Ser-536, which ultimately enhances the transcription of NF-κB and NFATc1 (16498455). Qian etc. reported that p38α deficiency induces increased bone mass in young mice, with decreased numbers of osteoclasts and bone resorption [34]. Consistently, it is reported that the treatment of p38 inhibitor ameliorated osteoclastogenesis [35]. Moreover, it has been demonstrated that the specific ablation of p38α in monocytic osteoclast precursors inhibited osteoblast proliferation and differentiation, indicating the critical role p38 played in coupling osteoclast and osteoblast function [34]. Our RNA-seq data indicated the involvement of MAPK signaling pathway with the Micheliolide treatment in osteoclasts (Figure 5A–5D). WB results further confirmed that p38 MAPK signaling, but not extracellular signal-regulated kinase (ERK) or c-Jun N-terminal kinase (JNK), was dramatically suppressed by Micheliolide treatment during osteoclastogenesis (Figure 5E). Moreover, the treatment of P79350, a kind of p38 agonist, successfully rescued the inhibitory effects caused by Micheliolide during osteoclastogenesis (Figure 6). Together, these data confirmed that Micheliolide manipulated osteoclast via inhibiting p38 MAPK signaling.

In addition, we should note that there is a certain limitation cannot be ignored in our study. The inhibitory effect and underlying mechanism of Micheliolide on osteoclast differentiation through inhibiting p38 signaling had been primarily explored, but the specific target of Micheliolide on osteoclast differentiation remains unclear, it is necessary to perform further in-depth study to identify the binding substances of Micheliolide for better understanding its role in osteolastogensis.

In conclusion, our current study demonstrated that Micheliolide attenuated pathological bone loss via inhibiting osteoclastogenesis. Mechanically, Micheliolide exerted this effect via restraining the activation of p38 MAPK signaling pathway during osteogenesis. Hence, we conclude that Micheliolide might could be used as an anti-resorptive agent against osteoporosis.

## MATERIALS AND METHODS

### Mice and reagents

Micheliolide was gained from MedChemExpress (USA). P79350 was purchased from Calbiochem (USA). CCK-8 kit and TRAP staining kit were purchased from Sigma-Aldrich (USA). TRIZOL was gained from Invitrogen (USA), reverse transcript reagents and SYBR Green PCR Master Mix were purchased from TaKaRa Biotechnology (Shiga, Japan). Antibodies for ERK, P38, JNK, P-ERK, P-P38, P-JNK, GAPDH were gained from Cell Signaling Technology (USA). Cytokines including RANKL and M-CSF were purchased from R&D system (USA). ELISA kits of CTX-1 and P1NP were obtained from IDS (UK). Wild type female C57/BL6 mice were purchased from the Shanghai Slake Experimental Animal Company (Shanghai, China).

### RNA sequencing analysis

For transcriptome RNA-seq analysis, BMMs were plated at the same density of 2.5*103 cell/cm in 4 different wells. 2 wells received DMSO treatment (Con-1, Con-2) and another 2 wells received 20 uM Micheliolide (MCL-1, MCL-2) treatment in the presence of M-CSF and RANKL for 5 days. Then, cells were collected and the total RNAs were isolated using Trizol reagent and subjected to RNA sequencing by Novogene Company (Beijing, China). Subsequent data analysis was conducted by RStudio and GSEA software. The raw sequence data reported in this paper have been deposited in the Genome Sequence Archive in National Genomics Data Center, China National Center for Bioinformation/Beijing Institute of Genomics, Chinese Academy of Sciences (PRJCA0123248) that are publicly accessible at https://ngdc.cncb.ac.cn/gsa [36, 37].

### Micro-CT scanning and analysis

The right femurs from each mouse were collected and the proximal femurs were collected and scanned by a Scanco μ-CT50 instrument (Scanco Medical, Brüttisellen, Switzerland) with 100 kV and 98 μA. The resolution was set to 10.5 μm and the region at a distance of 0.5 mm below the growth plate which was considered as the region of interest (ROI) for analysis. The three-dimensional (3D) reconstructions of the proximal femurs were conducted using the build-in software. The quantification data including BMD (mg HA/ccm), BV/TV (%), SMI, Tb.N (1/mm), Tb.Th (mm), and Tb.Sp (mm) were also analyzed using the built-in software (20533309 24780879 34801609).

### Histological analysis

Left femurs from each mouse were collected, dehydrated with gradient alcohol, infiltrated with a mixed solution of methyl methacrylate, dibutyl phthalate and benzoyl peroxide and finally embedded in methyl methacrylate. 5 μm sections were then cut along the coronal plane of the femur. The von Kossa staining of each sample was performed to evaluate BV/TV. The TRAP staining of the sections was also performed and the multinucleated TRAP positive cells were considered as osteoclasts. The bone resorption parameters including Oc.S/BS (%) and N.Oc/BS (1/mm) were then evaluated. Toluidine Blue staining was conducted to analysis osteoblast related parameters including N.Ob/BS and Obs/BS. Moreover, bone formation related parameters including MAR and BFR/BS were quantified under fluorescence microscopy.

### CTX-1 and P1NP bone turnover analysis

The blood from mice in each group was collected and serum levels of CTX-1 and P1NP were measured using the ELISA kit purchased from IDS according to manufacturer's instructions.

### Construction of OVX mouse model

Fifteen wild type female C57/BL6 mice aged 8 weeks were obtained and randomly split into three groups (n=5): Sham group (with Sham surgery and following with vehicle treatment), OVX group (with OVX surgery following with vehicle treatment), Micheliolide group (with OVX operation following with Micheliolide treatment (30 mg/kg/day)). Firstly, mice were anesthetized and received sham operation or OVX surgery. Then, mice in “Sham group” and “OVX group” were received vehicle (distilled water, 1 ml/kg/day) while mice in “Micheliolide group” were undertaken Micheliolide treatment (30 mg/kg/day) by oral administration. After 6 weeks, all mice were sacrificed and tibias were collected and fixed in 70% ethyl alcohol for further study. Of note, serum from each mouse was also collected, sending for bone metabolism ELISA assays.

### Cell culture

Bone marrow from 6 weeks old mice were flushed, plated on non-treated plates together with M-CSF for 72 hours. Then, BMMs were digested, counted and replated in wells in a density of 2.5*10^3^ cell/cm^2^. In order to induce osteoclast differentiation, BMMs were given 30 ng/ml M-CSF and 10 ng/ml RANKL treatment for 5 days. Then, cells were fixed and underwent TRAP staining using TRAP staining kit according to manufacturer's instructions. The number and area of osteoclasts (TRAP positive multinuclear cells) were counted using ImageJ software [38].

### CCK-8 cell viability analysis

The BMMs were seeded in 96-well plates at a density of 1.5*10^4^ cells/well and treated with M-CSF for 24 hours. After that, these cells were treated with vehicle (DMSO) or different concentrations of Micheliolide (5uM, 10uM, 20uM, 30uM, 40uM, 50uM) together with M-CSF for another 48 or 96 hours. The CCK-8 kit from Sigma-Aldrich was then used to evaluate the cell viability according to manufacturer's instructions.

### Pit formation assays

First of all, BMMs from 6 weeks old mice were collected and reseeded on collagen-coated plates, culturing for 5 days to get mature osteoclasts. After that, these mature osteoclasts were digested and seeded on dentin slice in a density of 1000 cells/96-well, culturing in the presence of different dosages of Micheliolide (5ug/ml, 10ug/ml, 20ug/ml) or vehicle (DMSO) for another 48 hours. Then, these dentin slices were collected, washed with PBS, followed by pit lection staining. The bone resorption area (brown staining area was considered as the resorption area) was then quantified using ImageJ software [38].

### RNA isolation and quantitative RT-PCR

Quantitative RT-PCR was conducted as described in our previous studies [2]. In short, BMMs were cultured in the presence of M-CSF and RNAKL together with DMSO or different dose of Micheliolide (5uM, 10uM, 20uM) for 5 days. Then total RNA was isolated using TRIZOL, cDNA was then synthesized and RT-PCR was performed. Mouse *Gapdh* was considered as the housekeeping gene and all the primer sequences used in this paper were described as follows: *Ctsk*, Forward 5′-AGGCATTGACTCTGAAGATGCT-3′, Reverse 5′-TCCCCACAGGAATCTCTCTG-3′; *Trap*, Forward 5′-CTGGAGTGCACGATGCCAGCGACA-3′, Reverse 5′-TCCGTGCTCGGCGATGGACCAGA-3′; *Gapdh*, Forward: 5′-TGCACCACCAACTGCTTAG-3′, Reverse: 5′-GGATGCAGGGATGATGTTC-3′; *DC-stamp*, Forward 5′-GGGGACTTATGTGTTTCCACG′, Reverse 5′-ACAAAGCAACAGACTCCCAAAT-3′; *C-fos*, Forward 5′-CCAGTCAAGAGCATCAGCAA-3′, Reverse 5′-AAGTAGTGCAGCCCGGAGTA-3′; *Nfatc1*, Forward 5′-CCGTTGCTTCCAGAAAATAACA-3′, Reverse 5′-TGTGGGATGTGAACTCGGAA-3′.

### Western blot analysis

BMMs were collected from 6 weeks old mice, cultured for 3 days, and then replated in 6-well plates at a density of 2.5*10^3^ cell/cm^2^. Then these cells received treatment of Micheliolide (20uM) or vehicle (DMSO) for 3 hours together with M-CSF, followed by the stimulation of RANKL (10 ng/ml) for indicated times (0, 5, 15, 30, 60 min). For the rescue study, BMMs received treatment of Micheliolide (20uM) or vehicle (DMSO) for 3 hours together with M-CSF, followed by the treatment of vehicle or P79350 (50 mmol/L) for 30min. The proteins of these samples were collected and the concentrations were measured using BCA protein kit. After that, same amounts of proteins from each sample (10 mg) were loaded in gel, being transferred to PVDF membranes. After being blocked in 5% milk for 2 hours, the membranes were then incubated with different primary antibodies at 4° for 16 hours. Then, these membranes were washed with TBST, incubated with secondary antibodies for another 1 hour. After that, the signaling of the proteins were detected using electrochemical luminescence reagent (ECL).

The antibodies to the MAPK in our study were provided by Abmart (Shanghai, China): p38 (T40075, 1:1000), p-p38 (T40076, 1:1000), ERK (T40071, 1:1000), p-ERK (T40072, 1:1000), JNK (T40073, 1:1000), p-JNK (TA3318, 1:1000). The antibody to GAPDH (60004-1-Ig, 1:5000) in our study was provided by Proteintech (Wuhan, China).

### Statistical analysis

All the *in vivo* and *in vitro* data were represented as mean ± standard deviation (SD). All the experiments were independently repeated at least three times. The difference between two groups were calculated by unpaired t-test and the difference between more than three groups were calculated by one-way ANOVA. Statistical significance was set as follows: *P<0.05, ^#^P<0.05, **P <0.01, ^##^P <0.01.
